# A transdermal treatment with MC903 ameliorates diet-induced obesity by reducing visceral fat and increasing myofiber thickness and energy consumption in mice

**DOI:** 10.1186/s12986-023-00732-5

**Published:** 2023-02-11

**Authors:** Tsutomu Wada, Yuichiro Miyazawa, Misa Ikurumi, Kento Fuse, Akira Okekawa, Yasuhiro Onogi, Shigeru Saito, Hiroshi Tsuneki, Toshiyasu Sasaoka

**Affiliations:** 1 Department of Clinical Pharmacology, University of Toyama, 2630 Sugitani, Toyama, 930-0194 Japan; 2 Research Center for Pre-Disease Science, University of Toyama, Toyama, Japan; 3 Department of Obstetrics and Gynecology, University of Toyama, Toyama, Japan; 4 Department of Integrative Pharmacology, University of Toyama, Toyama, Japan

**Keywords:** Calcipotriol, Myotube, Obesity, Transdermal treatment, Uncoupling protein 3 (UCP3)

## Abstract

**Aim:**

MC903 is a synthetic derivative of vitamin D3 that has been designed to diminish its impact on calcium metabolism and is clinically used as a transdermal reagent for psoriasis. Animal studies showed that an oral or intraperitoneal vitamin D3 treatment prevented the development of obesity. In contrast, the bioavailability of orally administered vitamin D3 is reported to be low in obese patients. In the current study, we aimed to investigate the impact of a transdermal treatment with MC903 in established obese mice. We further studied the underlying mechanisms of MC903-mediated metabolic improvement.

**Materials and methods:**

Male C57BL/6 J mice were fed standard chow or a 60% high-fat diet (HFD) for 7 weeks, and a transdermal treatment with MC903 on the ear auricle was initiated thereafter. The metabolic profiles of mice were analyzed during 4 weeks of treatment, and mice were dissected for histological and gene expression analyses. The direct impacts of MC903 and vitamin D3 were investigated using 3T3-L1 adipocytes and C2C12 myotubes in vitro.

**Results:**

HFD-fed mice showed significant increases in body and epididymal white adipose tissue (eWAT) weights with enlarged adipocytes. They exhibited glucose intolerance, decreased oxygen consumption, and chronic inflammation in eWAT. The transdermal treatment with MC903 significantly ameliorated these metabolic abnormalities in HFD-fed mice without affecting food consumption. In accordance with enhanced energy metabolism, myofiber diameters and the expression of uncoupling protein 3 (*UCP3*) in the gastrocnemius and soleus muscle were significantly increased in MC903-treated HFD mice. In addition, vitamin D3 and MC903 both suppressed adipogenic differentiation and enhanced lipolysis in 3T3-L1 adipocytes, and increased *UCP3* expression in cultured C2C12 myotubes. Furthermore, MC903 increased oxygen consumption and UCP3 knockdown significantly decreased them in C2C12 myotubes.

**Conclusions:**

A transdermal treatment with MC903 increased myofiber diameter and energy metabolism and decreased visceral fat accumulation, thereby improving obesity and glucose intolerance in mice.

**Supplementary Information:**

The online version contains supplementary material available at 10.1186/s12986-023-00732-5.

## Introduction

The prevalence of obesity and type 2 diabetes is increasing worldwide [1]. The excessive accumulation of nutrients causes the hypertrophy of adipocytes in obese visceral adipose tissue. These enlarged adipocytes produce reactive oxygen species, and adipose tissue becomes hypoxic due to the insufficient development of vessels, which promotes the infiltration and activation of immune cells, such as macrophages [2–4]. Chronic inflammation promotes systemic insulin resistance and associated metabolic abnormalities through aberrantly secreted inflammatory cytokines and adipocytokines [5]. In addition, these unfavorable mediators affect the hypothalamus to suppress energy metabolism, resulting in the further promotion of obesity [6, 7]. Therefore, effective interventions are required to suppress the various comorbidities associated with obesity and type 2 diabetes.

Psoriasis is a common chronic inflammatory skin disease that is characterized by epidermal hyperplasia and hyperkeratosis. It is triggered by genetic and environmental factors, including psychological stress, and immunological disturbances in tumor necrosis α (TNFα) and the IL-23/ IL-17 pathways play a role in its pathology [8, 9]. In addition, increased body weight and fatty acid intake are considered to be major risk factors for psoriasis. In this context, epidemiological studies have shown that the incidence of psoriasis is gradually increasing with obesity [10, 11]. In addition, recent cohort studies showed a relationship between the severity of psoriasis and the development of type 2 diabetes [12, 13].

Vitamin D3 (VitD3) is a functional nutrient that primarily contributes to the maintenance of bone and calcium homeostasis, and is also known to have pleiotropic biological functions [14]. VitD3 exerts immunomodulatory functions on various immune cells, such as T cells, dendritic cells, and macrophages to suppress inflammation [15, 16]. In addition, the seasonal exacerbation of psoriasis due to decreases in serum VitD3 levels associated with fluctuations in daylight hours has been reported [17]. In this context, VitD3 contributes to the maintenance of skin homeostasis with its anti-inflammatory function [9, 18]. Therefore, VitD3 derivatives are widely used as external medicine for psoriasis, alone or in combination with glucocorticoids, such as betamethasone dipropionate. MC903, calcipotriol, is a clinically used VitD3 derivative that is designed to diminish its impact on calcium and phosphate metabolism [19, 20]. Topical treatments have been shown to effectively suppress keratinocyte proliferation and skin inflammation in patients with psoriasis.

Furthermore, the biological function of VitD3 is suggested to be associated with glucose metabolism. Epidemiological studies revealed a relationship between low serum VitD3 levels and the risk of type 2 diabetes [21–23]. Moreover, the oral or intraperitoneal administration of VitD3 to mice prevented obesity and the associated impairment of glucose metabolism by suppressing chronic inflammation in visceral adipose tissue and the liver and by promoting beta cell function [24–26]. However, the effects of a transdermal treatment with VitD3 on established obesity and the associated impairments of glucose and energy metabolism remain unclear.

Therefore, we herein investigated the impact of a topical MC903 treatment on mice with diet-induced obesity. A transdermal treatment with MC903 on the ear auricle significantly reduced body weight and improved glucose metabolism in diet-induced obese mice. Mechanistically, MC903 increased skeletal muscle volumes, myofiber thicknesses, and the expression of *uncoupling protein* (*UCP*) 3 in lower limb muscles and enhanced oxygen consumption (VO_2_). The treatment also decreased obesity-associated chronic inflammation in visceral adipose tissue. Furthermore, VitD3 and MC903 both increased *Ucp3* expression in cultured myotubes. These results indicate the potential of a transdermal treatment with VitD3 as a novel therapeutic option for obesity and type 2 diabetes.

## Materials and methods

### Animals and the experimental protocol

**Fig. 1:**
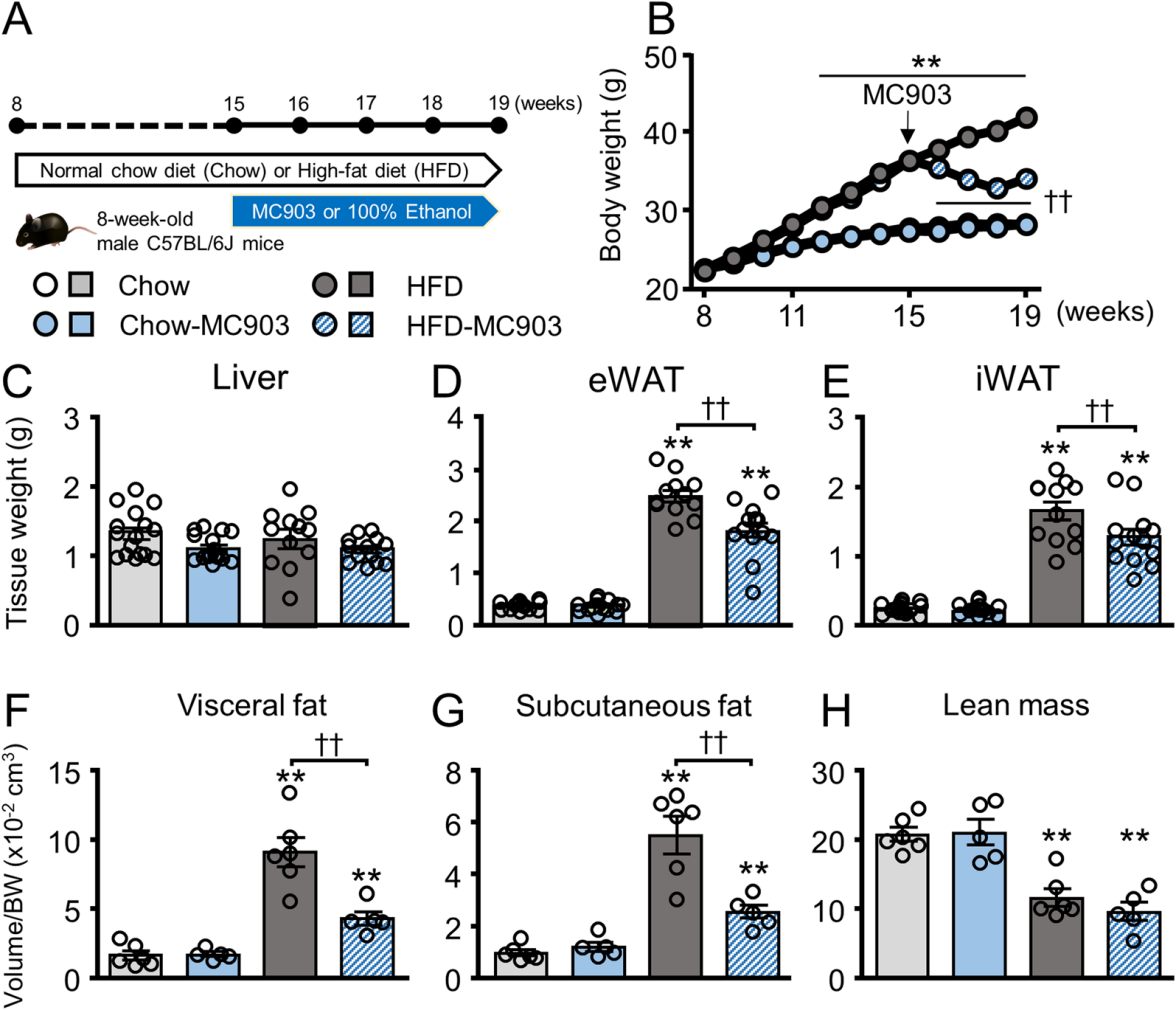
The transdermal MC903 treatment attenuated body weight and fat accumulation in HFD mice. **A** Experimental protocol of the study. **B** Transition of body weights. **C**-**E** Tissue weights at dissection. **F**-**H** Volumes of visceral fat, subcutaneous fat, and lean mass in 18-week-old mice analyzed by MRI. Data are presented as the mean ± SEM. N = 10–16 (**B**-**E**) or 5–6 (**F**–**H**). **p < 0.01 significantly different from Chow mice; ^†^*p* < 0.01 significantly different from HFD mice

Eight-week-old male C57BL/6 J mice (Japan SLC, Shizuoka, Japan) were fed a normal chow diet (Chow; Rodent Diet 20 5053; LabDiet, St. Louis, MO, USA) or a 60 kcal% high-fat diet (HFD; D12492; Research Diets, New Brunswick, NJ, USA). A transdermal treatment with MC903 (Bio-Techne, Japan) or vehicle was initiated at the age of 15 weeks. By using micropipettes, 20 μL of 200 μM MC903 dissolved in 100% ethanol or vehicle (100% ethanol) was applied to the auricle of mice 3 times a week for 4 weeks, yielding the following four mouse groups: Chow (N = 16), Chow-MC903 (N = 12), HFD (N = 16), and HFD-MC903 (N = 13) (Fig. 1A). MC903 solution immediately dries after application and does not crystallize. Licking of the application site by other mice in the same cage was not observed. The weekly external dose of MC903 per body weight is calculated to be nearly twice the optimal dose in humans, but is not considered a pharmacological dose because mice have a higher metabolic rate than humans. No obvious local skin damage, including dermatitis at the application site, and systemic toxic effects due to overdose of VitD3, such as diarrhea and kidney stones were observed in these mice (data not shown). Energy and glucose metabolism and body composition were analyzed during the MC903 treatment, and mice were dissected under anesthesia following the deprivation of food overnight for further analyses. Mice were housed under a 12:12-h light–dark cycle (lights on at 07:00) in a temperature-controlled colony room, and were provided food and water ad libitum. All experimental procedures used in the present study were approved by the Committee of Animal Experiments at the University of Toyama (Approval Number: A2017-PHA-1 and A2020-PHA-6).

### Analysis of body composition

The body fat composition of 18-week-old mice and lower limb muscle volume of 19-week-old mice were analyzed by magnetic resonance imaging (MRI) (MRmini SA, DS pharma Biomedical, Osaka, Japan) under anesthesia [27, 28]. Horizontal T1-weighted MRI cross-section from the diaphragm to the anus and from the hip joint to the distal end of the tibia was taken at 2 mm thickness. The volumes of visceral and subcutaneous adipose tissues, lean mass and lower limb muscles were obtained from the sum of each area in each slice × 2 mm, using the software ImageJ (National Institutes of Health, Bethesda, MD).

The volumes of visceral and subcutaneous adipose tissues and lower limb muscles were analyzed on images from the diaphragm to the anus and from the hip joint to the distal end of the tibia, respectively, using the software ImageJ (National Institutes of Health, Bethesda, MD). Tissue volumes were expressed after weight corrections.

### Glucose and insulin tolerance tests

The glucose tolerance test (GTT) was conducted by an intraperitoneal injection of glucose (2 g/kg body weight) after 6 h of fasting, and the insulin tolerance test (ITT) by an intraperitoneal injection of insulin (0.75 U/kg body weight) after 4 h of fasting, as previously described [29]. Each examination was performed on 17-week-old mice.

### Measurement of serum vitamin D, insulin, and calcium levels and the hepatic triglyceride content

Blood samples were collected from the abdominal aorta under anesthesia at the timing of dissection in the fasted state. Blood samples were centrifuged at 2,000 rpm for 20 min, and the resulting supernatants were subjected to measurements using 25-hydroxy-vitamin D3 ELISA that does not cross-react to any vitamin D analogue (Cloud-clone, the Netherlands), mouse insulin ELISA (Fujifilm, Japan), and a colorimetric calcium assay kit (Metallogenics, Japan). The hepatic triglyceride content was assessed using a triglyceride colorimetric kit (Wako Pure Chemical) after the extraction of the lipid fraction from frozen liver specimens [30]. These analyses were conducted in duplicate. Interassay coefficients of variations were less than 10% in each analysis.

### Real-time quantitative PCR

RNA extraction, reverse transcription, and real-time PCR using SYBR green were performed as previously described [29, 30]. The relative expression of objective mRNA was calculated as a ratio to that of 18S ribosomal RNA. Primer sequences are listed in Table 1.

**Table 1:** Primer list

Genes	Forward primer	Reverse primer
*Emr1*	CTTTGGCTATGGGCTTCCAGTC	GCAAGGAGGACAGAGTTTATCGTG
*Fbxo32*	CTTTCAACAGACTGGACTTCTCGA	CAGCTCCAACAGCCTTACTACGT
*Fndc5*	ATGAAGGAGATGGGGAGGAA	GCGGCAGAAGAGAGCTATAACA
*Igatx*	ATGTTGGTGGAAGCAAATGG	CCTGGGAATCCTATTGCAGA
*Il6*	ATGGATGCTACCAAACTGGAT	TGAAGGACTCTGGCTTTGTCT
*Mstn*	CTGTAACCTTCCCAGGACCA	TCTTTTGGGTGCGATAATCC
*Myf5*	TGAGGGAACAGGTGGAGAAC	AGCTGGACACGGAGCTTTTA
*Myhc1*	CTCAGGTGGCTCCGAGAAAG	TGGCTGAGCCTTGGATTCTC
*Myhc2a*	GCAAGAAGCAGATCCAGAAAC	GGTCTTCTTCTGTCTGGTAAGTAAGC
*Myhc2x*	GCAACAGGAGATTTCTGACCTCAC	CCAGAGATGCCTCTGCTTC
*Myhc2b*	TCTGGTAACACAAGAGGTGC	AAAAGGCTTGTTCTGGGCCT
*Myod1*	GCTGCCTTCTACGCACCTG	GCCGCTGTAATCCATCATGC
*Myog*	CCAACCCAGGAGATCATTTG	ACGATGGACGTAAGGGAGTG
*Pgc1a*	GCCCGGTACAGTGAGTGTTC	CTGGGCCGTTTAGTCTTCCT
*Pparg*	TCGCTGATGCACTGCCTATG	TGTCAAAGGAATGCGAGTGGTC
*Tnfa*	AGCCTGTAGCCCACGTCGTA	GGCACCACTAGTTGGTTGTCTTTG
*Trim63*	GGACTACTTTACTCTGGACTTAGAAC	CAGCCTCCTCTTCTGTAAACTC
*Ucp1*	TACCAAGCTGTGCGATGT	AAGCCCAATGATGTTCAGT
*Ucp3*	ATCGCCAGGGAGGAAGGA	GTTGACAATGGCATTTCTTGTGA
*Vdr*	CACGGGCTTCCACTTCA	CGAGCAGGATGGCGATAA
*18 s rRNA*	GTAACCCGTTGAACCCCATT	CCATCCAATCGGTAGTAGCG

### Histological analysis and immunohistochemistry

Isolated epididymal white adipose tissue (eWAT) and livers were fixed in 4% formaldehyde for 24 h and embedded in paraffin. Six-micrometer-thick sections were stained with hematoxylin & eosin (H&E) and then used in subsequent analyses. Regarding CD11c immunohistochemistry, paraffin-embedded sections were incubated with a hamster anti-mouse CD11c antibody (dilution 1:100, 10 μg/mL) for 3 h followed by a goat anti-hamster IgG antibody (dilution 1:100, 8 μg/mL) for 1 h. Photomicrographs were captured using the microscope BX61 (Olympus, Tokyo, Japan). Isolated gastrocnemius muscles were quenched with hexane and frozen. Tissue was embedded in OCT compound (Sakura Finetek, Osaka, Japan), and 20-µm-thick cross-sections were obtained from the center of muscle tissue using a cryostat and stained with H&E. Photomicrographs were captured using BZX800 (Keyence, Osaka, Japan). The cross-sections of each adipocyte and myofiber were traced one by one, and the area of each traced region was analyzed, using ImageJ 1.45 s software (NIH) or the BZX800 system (Keyence), respectively [29, 31]. The average size of adipocytes and myofiber was calculated by analyzing ~ 300 adipocytes or myofibers per mouse from the H&E-stained sections of eWAT or gastrocnemius muscle.

### Analysis of energy metabolism

Oxygen consumption (VO_2_), carbon dioxide production (VCO_2_), and locomotor activity in 18-week-old mice were analyzed using metabolic chambers (MK-5000RQ, Muromachi Kikai, Tokyo, Japan) with free access to food and water, as previously described [27, 32].

### Differentiation of 3T3-L1 adipocytes, oil red O staining and lipolysis analysis

3T3L1 preadipocytes were differentiated into mature adipocytes, as described previously [33]. In brief, differentiation was induced in confluent cells with differentiation medium containing 10% fetal bovine serum (FBS), 250 nM dexamethasone, 0.5 mM isobutyl methylxanthine, and 500 nM insulin. After 3 days, the differentiation medium was replaced with post-differentiation medium containing 10% FBS and 500 nM insulin. After 3 more days, post-differentiation medium was replaced with DMEM supplemented with 10% FBS. Cells were differentiated in the absence or presence of 300 nM MC903 or 1,25-dihydroxy-VitD3 (Cayman Chemical, USA). Cells were harvested every 3 days after initiation of differentiation and were subjected to a real-time PCR analysis. Photomicrograph of Oil-Red O stained 3T3-L1 adipocytes was taken at day 8 post-differentiation, as described previously [34].

For the lipolysis assay, 3T3-L1 adipocytes at day 5 post-differentiation were treated with 300 nM MC903 or 1,25-dihydroxy-VitD3 for 3 days. Cells were stimulated with 10 μM isoproterenol for 6 h, and glycerol level in the media was analyzed using a glycerol colorimetric assay kit (Cayman).

### Cultivation of C2C12 myotubes

C2C12 myoblasts were cultured in high glucose DMEM (Thermo Fisher Scientific) supplemented with 10% FBS. Two-day postconfluent myoblasts were induced to differentiate into myotubes using myotube differentiation medium composed of low glucose DMEM with 2% horse serum (Invitrogen) [35]. Cells were differentiated in the absence (control) or presence of 300 nM MC903 or 1,25-dihydroxy-VitD3 for 72 h, harvested, and subjected to a real-time PCR analysis.

### Knockdown of Ucp3 and oxygen consumption rate assay

C2C12 myoblasts seeded at 2 × 10^4^ cells/well in a Seahorse XFe24 assay plate (Agilent, USA) were incubated with DMEM supplemented with 10% FBS and antibiotics. After 24 h incubation, culture media were replaced to myotube differentiation medium. Further after 24 h, medium was replaced to Opti-MEM with 0.1% FBS, and cells were incubated with lipid complexes containing 0.25% Lipofectamine RNAiMAX and 50 nM of *Ucp*3 siRNA or scrambled siRNA control for 24 h. The siRNA sense sequence is 5′- GAUGUGGUAAAGACCCGAUACAUGA-3′. Then media were replaced to myotube differentiation medium and incubated for another 24 h. MC903 was added to the medium at 100 nM after initiation of differentiation in the treated cells. Then media were replaced with fresh Seahorse XF assay medium supplemented with 5 mM glucose and 1 mM pyruvate. After 1 h of incubation, oxygen consumption of the cells was measured using Seahorse XFe24 Analyzer (Agilent), according to the manufacturer’s instruction. Each treatment was injected sequentially to achieve the following final concentrations: 1.5 μM oligomycin (Biomol, Hamburg, Germany; #CM-111), 1 μM carbonyl cyanide-p-trifluoromethoxyphenylhydrazone (FCCP) (Biomol), 2 mM glutamine (Sigma), 0.5 μM rotenone (Rot) (Biomol) and 0. μM antimycin A (AA) (Sigma). The OCR measured after FCCP treatment was analyzed as the maximum OCR.

### Statistical analysis

Data are expressed as the mean ± S.E. Statistical analyses were performed using an unpaired two-tailed Student’s *t*-test between two groups and a one-way ANOVA followed by the Bonferroni test for multiple comparisons using the software ystat2004. *P* < 0.05 was considered to be significant.

## Results

### MC903 reduced body weights and fat accumulation in obese mice

Eight-week-old mice were maintained with standard chow or 60% HFD, and the MC903 treatment to the ear auricle was initiated when mice were 15 weeks old. The body weight transition in each group is shown in Fig. 1B. Increases in body weight were markedly higher in HFD mice than in Chow mice, and were significantly reduced by the transdermal MC903 treatment. In contrast, MC903 did not affect the body weight of Chow mice. In the tissue weight analysis, liver weight did not change among the four groups (Fig. 1C). Although the weights of eWAT and inguinal WAT (iWAT) were significantly increased by HFD, they were lower in HFD-MC903 mice than in HFD mice (Fig. 1D, E). Similarly, visceral and subcutaneous fat volumes were significantly lower in HFD-MC903 mice than in HFD mice (Fig. 1F, G). In contrast, lean upper body mass remained unchanged by the MC-903 treatment, but was higher in HFD mice than in Chow mice (Fig. 1H). Neither apparent skin damage nor dermatitis was observed with the M903 treatment (data not shown).

### MC903 improved glucose metabolism without affecting serum vitamin D and calcium levels

**Fig. 2:**
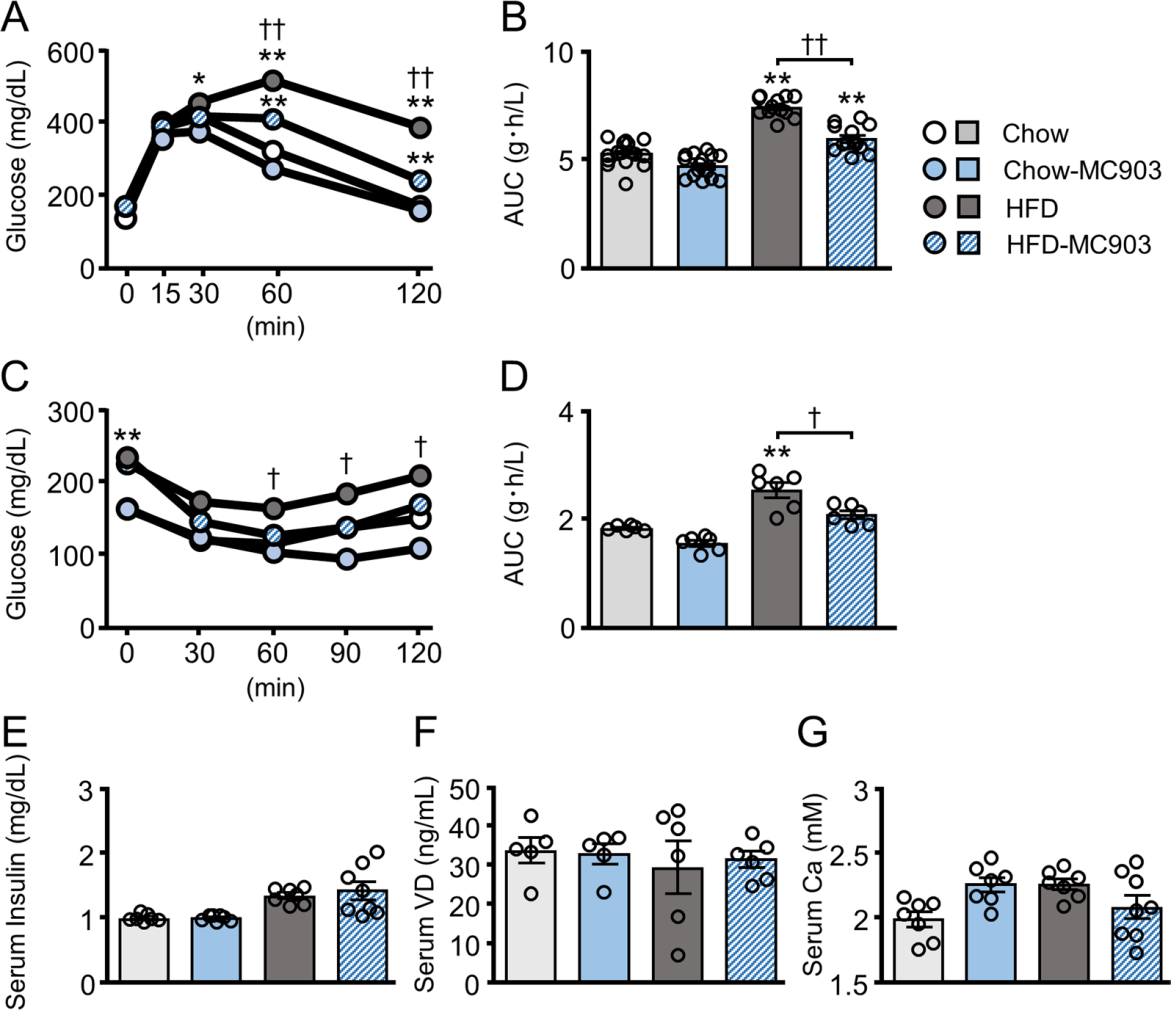
The transdermal MC903 treatment improved glucose metabolism without affecting serum VitD3 and calcium levels. **A**-**D** Blood glucose levels and their areas under the curve (AUC) in GTT and ITT. **E** Fasted serum insulin levels. **F, G** Serum 25-hydroxy-vitamin D3 and calcium levels at dissection. Data are presented as the mean ± SEM. N = 12–16 (**A**, **B**) or 5–8 (**C**-**G**). *p < 0.05, **p < 0.01 significantly different from Chow mice; ^†^*p* < 0.05, ^††^*p* <  0.01 significantly different from HFD mice

Since diet-induced fat accumulation in visceral adipose tissue is closely associated with glucose metabolism, GTT and ITT were performed on mice. Blood glucose levels and their area under the curve in both GTT and ITT were significantly higher in HFD mice than in Chow mice, and these increases in HFD mice were significantly ameliorated by the MC903 treatment (Fig. 2A-D). In contrast, fasted insulin levels did not change among the four groups of mice (Fig. 2E). Consistent with the properties of MC903 designed to diminish its impact on calcium metabolism [19], neither 25-hydroxy-vitamin D3 nor calcium levels were affected by the transdermal treatment with MC903 (Fig. 2F, G).

### MC903 attenuated the chronic inflammation of visceral adipose tissue

**Fig. 3:**
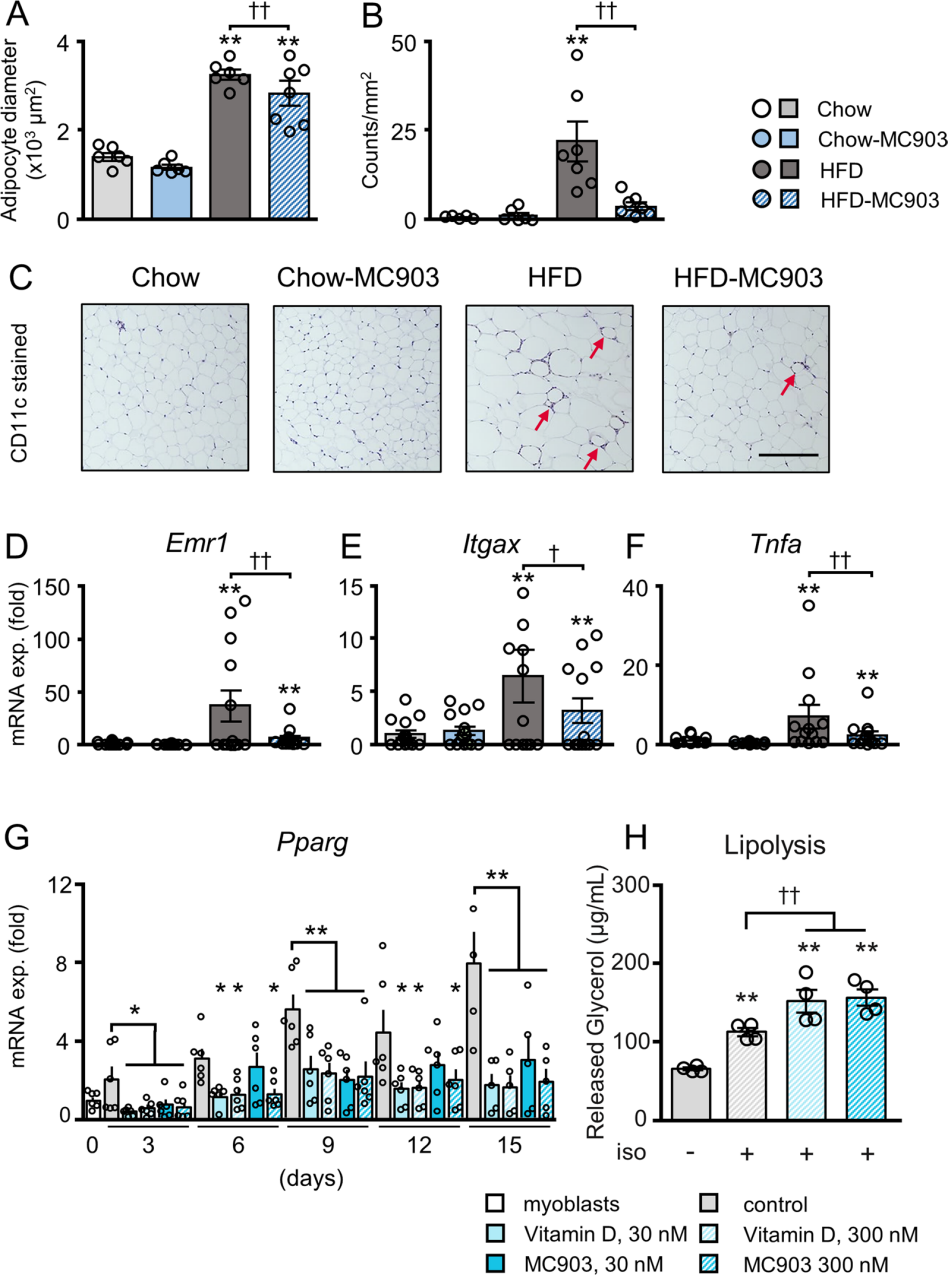
MC903 treatment attenuated adipocyte hypertrophy and chronic inflammation in eWAT. **A** Average size of adipocytes. **B**, **C** Quantified numbers of crown-like-structures (CLS) and representative photomicrographs of anti-CD11c immunostaining in eWAT. The arrow indicates CLS. **D**-**F** Gene expressions in eWAT. **G** Effect of VitD3 and MC903 on mRNA expression of *Pparg* in differentiating 3T3-L1 adipocytes. **H** Effect of VitD3 and MC903 on isoproterenol-induced glycerol release in 3T3-L1 adipocytes. Data are presented as the mean ± SEM. N = 5–7 (A-C, G), 13–15 (D-F), or 4 (H). *p < 0.05, **p < 0.01 significantly different from Chow mice; ^†^*p* < 0.05, ^††^*p* < 0.01 significantly different from HFD mice

Since chronic inflammation in visceral adipose tissue is a pathology of obesity that is closely associated with the development of insulin resistance and metabolic disturbances [2–5], we investigated the histological features and chronic inflammation of eWAT in each mouse. H&E staining of eWAT revealed that the average adipocyte size was significantly larger in HFD mice than in Chow mice, which was attenuated in HFD-MC903 mice. In contrast, MC903 did not affect average cell sizes in Chow mice (Fig. 3A, Additional file 1: Figure S1). In the size distribution analysis, HFD-fed mice showed an increase in large adipocytes, which was attenuated by the treatment with MC903. The number of adipocytes within 3,000 to 5,500 μm^2^ was higher while that of more than 9000 μm^2^ was lower in HFD-MC903 mice than in HFD mice, indicating that adipocyte hypertrophy was attenuated by the treatment. Similarly, the number of adipocytes of more than 2500 μm^2^ was lower in Chow-MC903 mice than in Chow mice (Additional file 1: Figure S1B, C). eWAT in HFD mice showed a significant increase in crown-like structures (CLS), a histological hallmark of chronic inflammation composed of accumulated CD11c-positive proinflammatory macrophages surrounding dysfunctional adipocytes, whereas eWAT in HFD-MC903 mice showed a significant decrease in these structures (Fig. 3B, C). The mRNA expression of the proinflammatory genes of *Emr1* encoding F4/80, the macrophage marker, *Itgax* encoding CD11c, an inflammatory macrophage marker, and *Tnfa* was significantly higher in HFD mice than in Chow mice, and was decreased in HFD-MC903 mice (Fig. 3D-F).

We investigated the impacts of VitD3 and MC903 on the differentiation and lipolytic activity in adipocytes in vitro. The expression of *Pparg* was significantly lower at all time points analyzed in 3T3-L1 adipocytes differentiated in the presence of VitD3 or MC903 (Fig. 3G). In addition, Oil Red O staining showed a trend toward decreased lipid accumulations in adipocytes differentiated in the presence of VitD3 or MC903 (Additional file 1: Figure S1G). Moreover, isoproterenol-induced glycerol release was significantly higher in adipocytes treated with VitD3 or MC903 (Fig. 3H). These in vitro results suggest the potential ability of VitD3 and its analogue to reduce lipid accumulations in adipose tissue by attenuating adipocyte differentiation and enhancing lipolysis*.*

### MC903 attenuated lipid accumulation in the liver

**Fig. 4:**
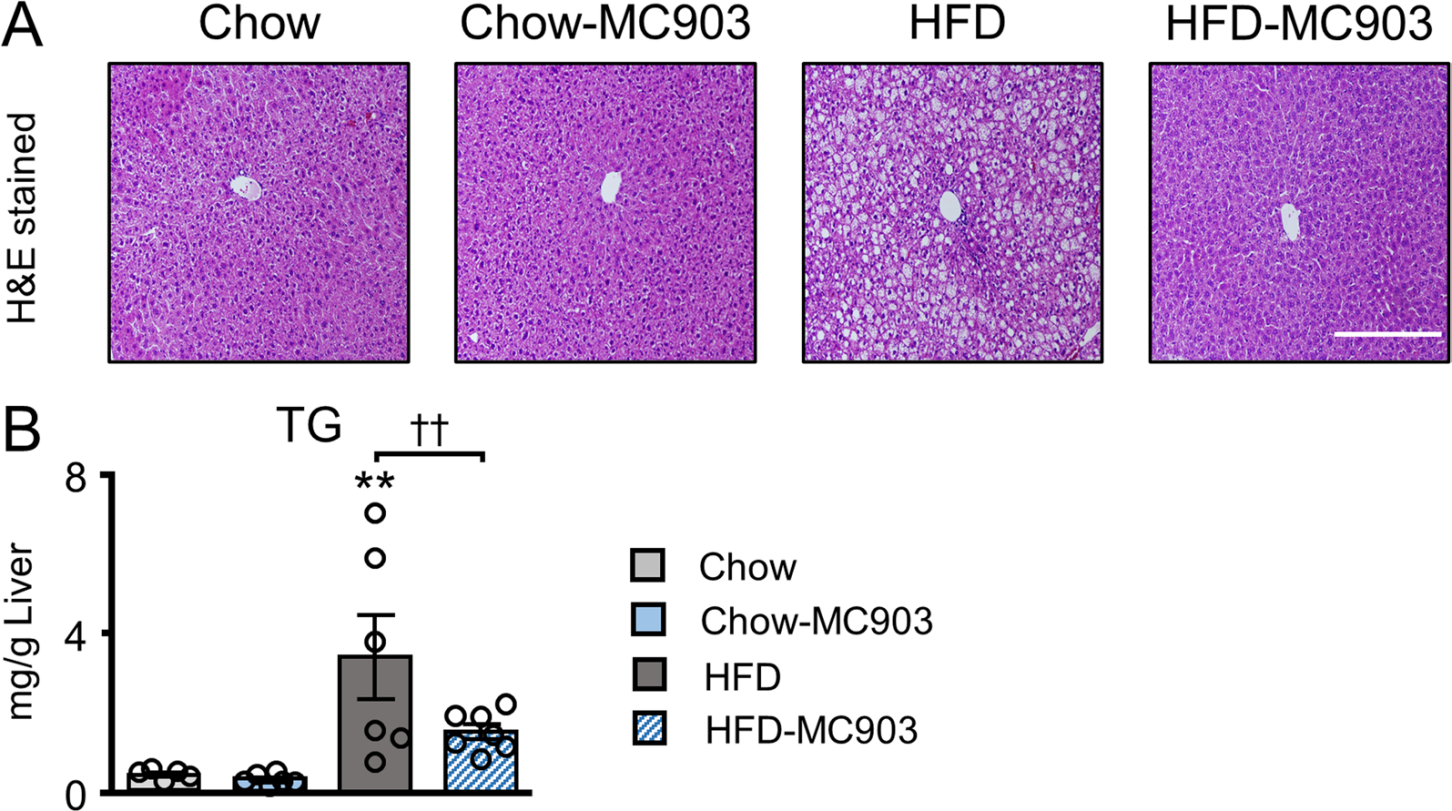
MC903 treatment attenuated lipid accumulation in the liver. **A** Representative photomicrograph of H&E-stained sections of the liver. **B** Triglyceride content in the liver. Scale bar, 200 μm. Data are presented as the mean ± SEM. N = 6–7. **p < 0.01 significantly different from Chow mice; ^††^*p* < 0.01 significantly different from HFD mice

We examined the impact of the transdermal treatment with MC903 on lipid accumulation in the livers of mice. In the histological analysis, the livers of HFD mice exhibited the typical histological features of hepatic steatosis, including significant lipid accumulation in hepatocytes and vacuole degeneration. In contrast, an almost normal liver structure was maintained with only mild fatty infiltration in the livers of HFD-MC903 mice (Fig. 4A). Consistent with these results, the triglyceride content in the liver was significantly higher in HFD mice, whereas it was significantly lower in HFD-MC903 mice than in HFD mice (Fig. 4B).

### MC903 improved energy metabolism in obese mice

**Fig. 5:**
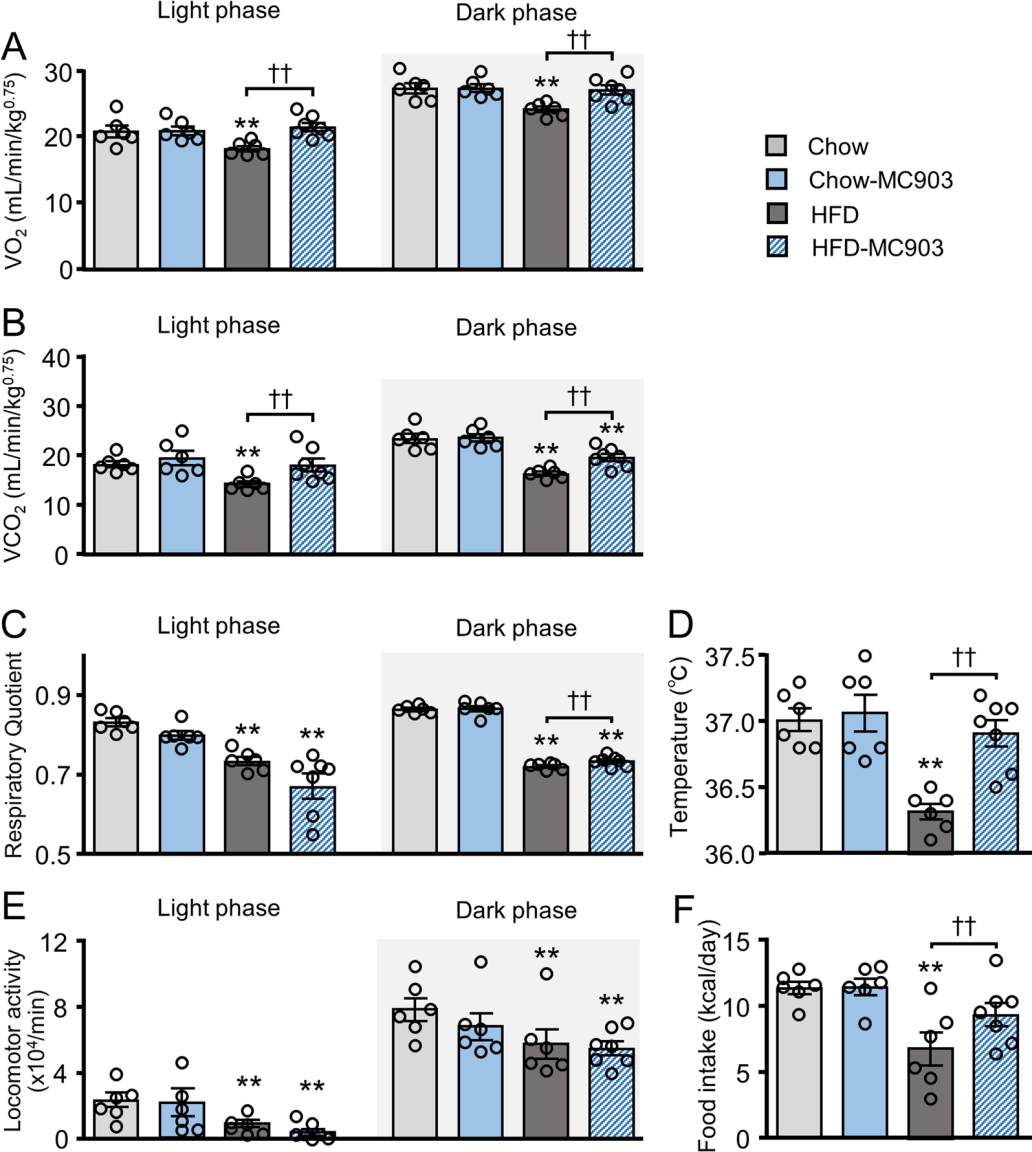
MC903 treatment enhanced energy metabolism in obese mice, but not in lean mice. **A** Oxygen consumption (VO_2_). **B** Carbon dioxide production (VCO_2_). **C** Respiratory Quotient. **D** Rectal temperature. **E** Spontaneous locomotor activity. **F** Daily food intake. Data are presented as the mean ± SEM. N = 6–7. **p < 0.01 significantly different from Chow mice; ^††^*p* < 0.01 significantly different from HFD mice

We investigated energy metabolism in each mouse to elucidate the underlying mechanisms for reductions in body weight and lipid accumulation in the livers of HFD-MC903 mice. VO_2_ and VCO_2_ in the light and dark phases were significantly lower in HFD mice than in Chow mice. Importantly, VO_2_ and VCO_2_ levels were significantly higher in HFD-MC903 mice than in HFD mice (Fig. 5A, B). In contrast, the MC903 treatment did not affect energy metabolism in lean mice. The respiratory quotient (RQ) was significantly lower in HFD and HFD-MC903 mice than in Chow mice and was close to 0.7, while RQ in the dark phase was significantly higher in HFD-MC903 mice than in HFD mice (Fig. 5C). Although core body temperature was significantly lower in HFD mice than in Chow mice, it recovered to almost an equivalent level in HFD-MC903 mice to that in Chow mice (Fig. 5D). Spontaneous locomotor activity was decreased in HFD mice in both phases, and was not affected by MC903 (Fig. 5E). Daily food intake was lower in HFD mice and higher in HFD-MC903 mice than in HFD mice, which appeared to be due to compensatory responses to increased energy expenditure (Fig. 5F).

### MC903 did not affect *UCP1* expression in BAT, but increased myofiber thickness and *UCP3* expression in muscles

Brown adipose tissue (BAT) and beige adipose tissue are heat-producing tissues that play crucial roles in the regulation of energy metabolism by catabolizing stored lipids to generate heat, whereas the accumulation of excess lipids in BAT during obesity affects their thermogenic activity [36, 37]. We analyzed histological changes and the expression of *UCP1*, a well characterized heat-producing effector in BAT and an indicator of beige adipocytes in iWAT, to examine the involvement of brown/beige adipocytes in enhanced energy metabolism in HFD-MC903 mice. The interscapular BAT of HFD mice exhibited a decrease in multilocular adipocytes and an increase in hypertrophic monocular adipocytes. In contrast, adipocytes were markedly smaller and their multilocular structure was maintained in the interscapular BAT of HFD-MC903 mice. Despite these histological changes, *UCP1* expression in BAT did not significantly change among the four groups of mice. In addition, neither beige adipocytes nor increased *UCP1* expression was observed in the iWAT of MC903-treated mice (Additional file 1: Figure S1F and data not shown). Therefore, the thermogenic activity of BAT and beige adipocytes did not play an important role in increasing energy expenditure in HFD-MC903 mice.

**Fig. 6:**
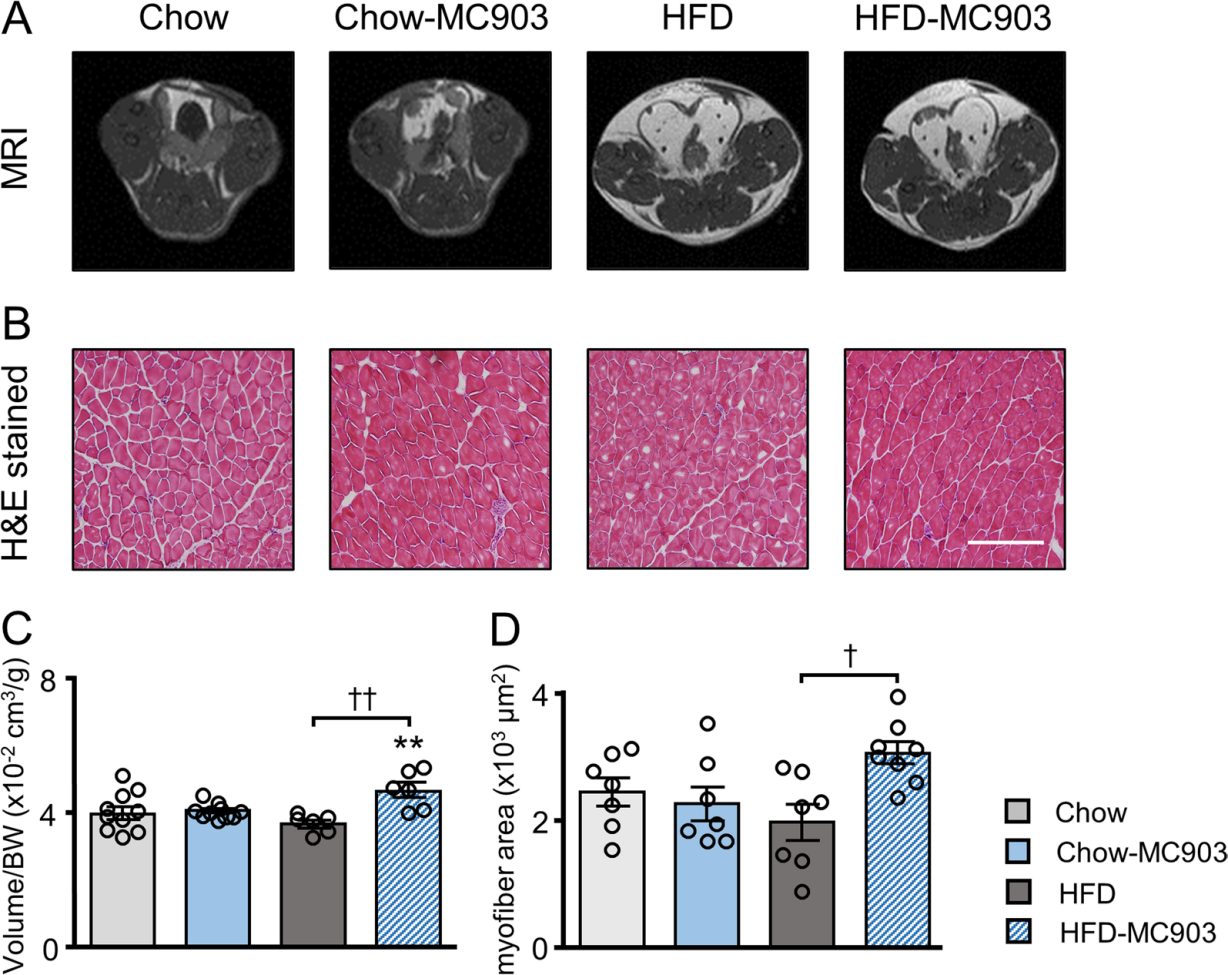
MC903 treatment increased muscle volumes and myofiber thicknesses in lower limbs of HFD mice. **A** Representative MRI images of the femoral region of mice. **B** Representative H&E-stained cross-sectional images of the gastrocnemius muscle. **C** Quantitative muscle volumes. **D** Average sizes of myofibers. Scale bar, 200 μm. Data are presented as the mean ± SEM. N = 7–10. **p < 0.01 significantly different from Chow mice; ^†^*p* < 0.05, ^††^*p* < 0.01 significantly different from HFD mice

Muscle is essential for the regulation of glucose and energy homeostasis. Since myocytes are a classical target of VitD3 [14], we examined muscle volumes in the lower limbs of each mouse using MRI. Muscle volumes were almost identical among Chow, Chow-MC903, and HFD mice. In contrast, they were significantly higher in HFD-MC903 mice than in HFD mice (Fig. 6A, C). We also investigated the cross-sectional area of muscle fibers in the gastrocnemius muscle in H&E-stained sections using the Keyence BZX-800 microscope system. Consistent with increased lower limb muscle masses, myofiber sizes were significantly larger in HFD-MC903 mice than in HFD mice (Fig. 6B, D).

**Fig. 7:**
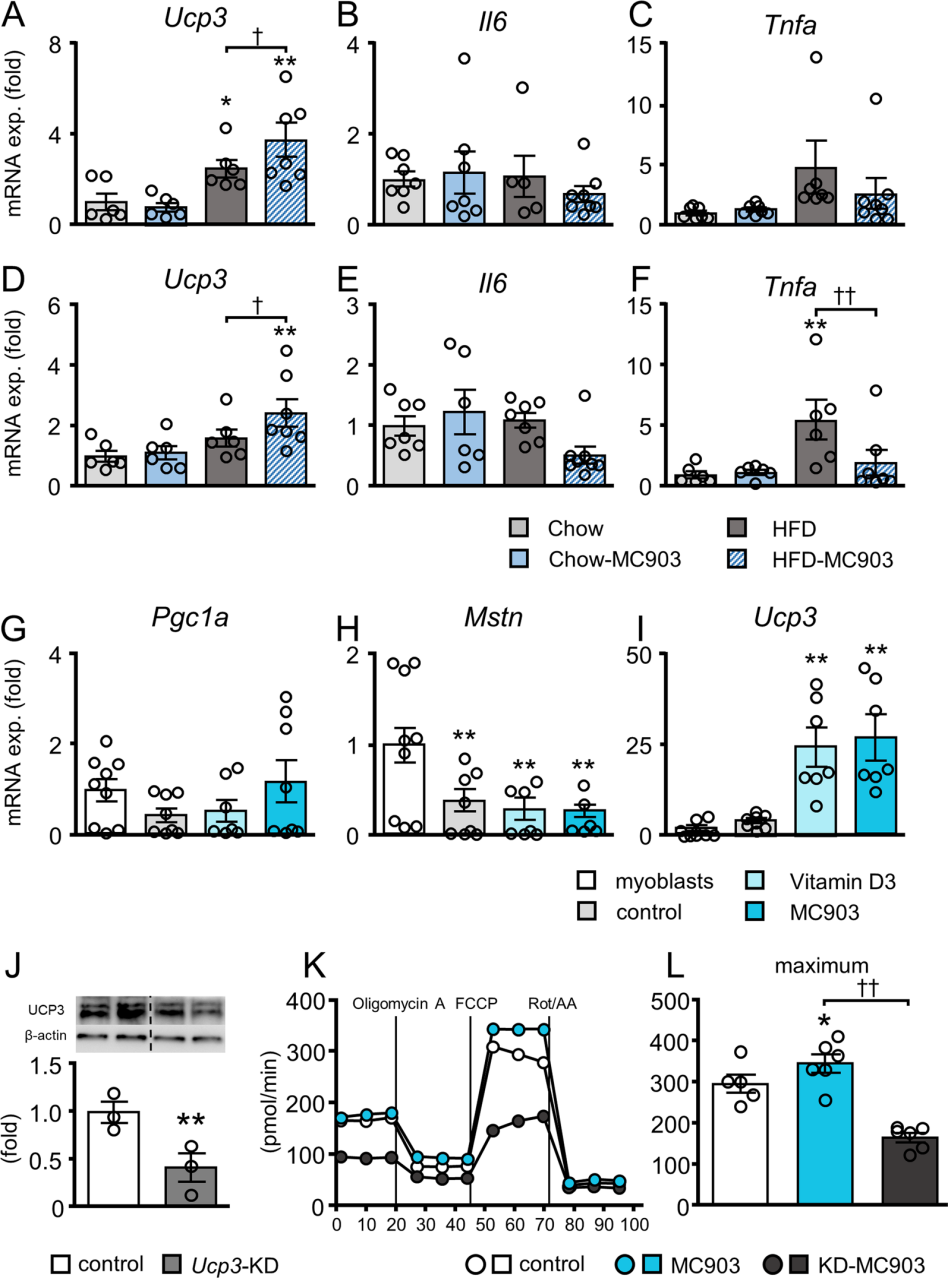
MC903 treatment increased *UCP3* expression in muscles in vivo and in vitro. **A**-**F** Gene expressions in the gastrocnemius (**A**-**C**) and soleus muscles (**D**-**F**). **G**-**I** C2C12 myoblasts were differentiated into myotubes in the absence (control) or presence of VitD3 or MC903, and the expression of *Pgc1a*, *Myostatin*, and *Ucp3* was analyzed. **J** UCP3 protein levels in knockdown myotubes. **K**, **L** OCR and maximum OCR in myotubes. Data are presented as the mean ± SEM. N = 6–9 (A-I), 3 (J) or 5–6 (K, L). **A**-**F** *p < 0.05, **p < 0.01 significantly different from Chow mice; ^†^*p* < 0.05, ^††^*p* < 0.01 significantly different from HFD mice. **G-L** *p < 0.05, **p < 0.01 significantly different from control; ^††^p < 0.01 significantly different from MC903-treated myotubes

UCP3 plays a significant role in muscle energy metabolism [38]. Notably, *UCP3* expression in both the gastrocnemius and soleus muscles was significantly higher in HFD-MC903 than in HFD mice (Fig. 7). In contrast, *Il6* expression was unchanged, while *Tnfa* expression was increased only in the soleus muscle of HFD mice and was decreased in HFD-MC903 mice. We also investigated the expression of *Fndc5* encoding irisin, genes associated with myocyte differentiation, muscle degradation-related ubiquitin ligase, muscle fiber types, and *vitamin D receptor* (*Vdr*). However, no significant changes were observed in the expression of these genes in the gastrocnemius and soleus muscles among the groups (Additional file 2: Figure S2, Additional file 3: Figure S3).

We examined the impact of VitD3 and MC903 in C2C12 myotubes to investigate the direct effects of MC903 on *UCP3* expression in muscle cells. The expression of *Pgc1a* did not significantly change between myoblasts and myotubes, whereas the level of *Mstn* encoding myostatin was significantly lower in control myotubes than in myoblasts during the differentiation process, which was consistent with previous findings [39] (Fig. 7G, H). The expression of these genes was not affected by treatment with VitD3 or MC903. In contrast, *UCP3* expression was significantly higher in VitD3- and MC903-treated myotubes than in control myotubes (Fig. 7I). We further examined the impact of UCP3 knockdown on oxygen consumption of these cells to examine whether MC903-induced increase of UCP3 directly affects cellular energy metabolism. The mRNA and protein level of UCP3 were decreased to 6.7 ± 0.4% and 39.2 ± 9.0% (Fig. 7J), respectively, by siRNA-mediated knockdown in myotubes. Importantly, maximum OCR was higher in MC903-treated myotubes than in untreated myotubes, which was significantly decreased by UCP3 knockdown (Fig. 7K, L).

## Discussion

The number of patients with obesity and sarcopenia is increasing due to the sedentary lifestyle of aging societies [40]. Obesity-associated insulin resistance promotes the development of various comorbidities, such as type 2 diabetes and cardiovascular diseases [5, 41]. The beneficial effects of VitD3 on glucose metabolism have been demonstrated in animal studies; however, its administration was simultaneously initiated with an obesogenic diet in most studies [25, 26]. In the present study, MC903 was transdermally applied to HFD-loaded established obese mice. MC903 increased energy metabolism, decreased body weight, and attenuated the distinctive pathology of obesity, such as insulin resistance, fat accumulation, chronic inflammation in the eWAT, and hepatic steatosis (Figs. 1, 2, 3, 4). Importantly, the transdermal treatment with MC903 increased muscle volumes, myofiber diameters, and *UCP3* expression in the lower limb muscles of HFD-fed mice (Figs. 5, 6). The expression of *UCP3* was increased by both VitD3 and MC903 in cultured C2C12 myotubes (Fig. 6). Since the bioavailability of VitD3 with its oral administration is decreased in obese patients [42], a transdermal treatment with MC903 may be a novel therapeutic strategy for obesity and type 2 diabetes.

Due to its anti-inflammatory effects on immune cells, VitD3 is used to treat psoriasis [20]. It has been shown to shift the macrophage polarity from the inflammatory to anti-inflammatory phenotype and suppress proinflammatory cytokine production by macrophages [43, 44]. In addition, severe chronic inflammation in the liver, impaired glucose metabolism, and the promotion of arteriosclerosis have been reported in myeloid-specific VDR knockout mice fed HFD [45]. Furthermore, vitamin D has been shown to attenuate differentiation and lipid accumulations in adipocytes [46]. Indeed, the formation of CLS, *Tnfa* expression and adipocyte size were significantly lower in visceral adipose tissue of HFD-MC903 mice than in HFD mice (Fig. 3, Additional file 1: Figure S1). Furthermore, MC903 inhibited adipogenic differentiation and enhanced lipolysis in 3T3-L1 adipocytes (Fig. 3G, H , Additional file 1: Figure S1). Since mitochondrial oxidation of fatty acids is the major source of energy production in skeletal muscle, the enhanced whole-body metabolism observed in HFD-MC903 mice may be induced by efficient consumption of adipose tissue-derived fatty acid in skeletal muscle.

Skeletal muscle is one of the major target tissues of VitD3, and its homeostatic significance for the prevention of obesity has been well characterized [14, 47]. The simultaneous administration of oral VitD3 to mice fed a high-fat high-sugar diet prevented body weight gain, lipid accumulation in muscles, and the deterioration of glucose metabolism [25]. Conversely, an increased muscle lipid content was detected in mice maintained on a low VitD3 diet [48]. More direct evidence for the effects of VitD3 on muscle function were provided from a series of VDR-deficient animals. However, the interpretation of the muscle phenotype in VDR-deficient mice requires careful attention because the congenital and systemic deletion of VDR affects the development and growth of mice, and the onset of hypocalcemia and cirrhosis has been reported [49]. The myofibers of systemic VDR-deficient mice with or without calcium corrections were found to be small [50, 51]. In addition, an enhanced ubiquitin–proteasome pathway was identified as the mechanism underlying muscle atrophy in VitD3 deficiency [52]. Moreover, the strength and volumes of muscles in muscle-specific VDR knockout mice were reduced due to decreases in myofiber numbers and their proliferation capacity [53]. Since myofiber diameters were increased in the gastrocnemius muscle of HFD-MC903 mice (Fig. 6), we examined the expression of genes related to muscle differentiation, namely, *Myod*, *Myog* encoding Miogenin, and *Myf5* and the proteasomal degradation genes *Fbxo32* encoding Atrogin1 and *Trem63* encoding Murf1 in the gastrocnemius and soleus muscles of each mouse. However, no significant changes were observed in their expression levels, at least under the current experimental conditions (Additional file 2: Figure S2, Additional file 3: Figure S3).

Tissue-specific differences in the impact of VitD3 on thermogenic gene expression have been reported. Serum calcium-corrected VDR-deficient mice showed high UCP1 expression levels in BAT [54]. In addition, VitD3 attenuated the differentiation of brown adipocytes in vitro [55], suggesting that VitD3 exerted negative effects on BAT thermogenesis. On the other hand, VitD3 has been reported to increase UCP3 expression in skeletal muscle both in vivo and in vitro [56]. In the present study, the expression of *UCP1* did not change in BAT, whereas that of *Ucp3* increased in the soleus and gastrocnemius muscles of HFD-MC903 mice (Fig. 7, Additional file 1: Figure S1). These results indicate that MC903 predominantly affected muscles rather than BAT to enhance energy metabolism in obese mice. We also examined the expression of the thermogenesis-related myokines *Il6* and *Fndc5* in these muscles [57]; however, no significant differences were observed among the groups. Regarding the effects of VitD3 in the hypothalamus, the chronic intracerebroventricular administration of VitD3 to HFD-fed mice was shown to suppress food consumption and body weight gain without affecting energy metabolism [58]. Therefore, the central effects of VitD3 differ from the phenotype in HFD-MC903 mice, which showed enhanced energy metabolism and food consumption (Fig. 5). Furthermore, the permeability of VitD3 to the central nervous system was previously shown to be low [58, 59]. Therefore, we considered that the increases induced in energy metabolism by the transdermal MC903 treatment appear to be due to its effects on muscles via the circulation rather than the direct activation of the hypothalamus. Indeed, we observed increased *Ucp3* expression and maximum OCR in MC903-treated C2C12 myotubes, which were decreased by UCP3 knockdown (Fig. 7). However, we were unable to measure serum MC903 levels in mice due to methodological limitations. Further studies are needed to examine the effects of MC903 using muscle-specific VDR-deficient mice.

Obesity-associated pathophysiology develops with connected to disruption of tissue-tissue interactions [5–7]. According to the Prescribing Information, MC903 immediately accumulates in the liver following external treatment, and is metabolized to inactive form. In contrast, the tissue distribution of VitD3 has been reported to be 20% in muscle, 30% in serum, 35% in fat, and 15% in all other tissues [60]. Therefore, it is assumed that MC903 may directly change the expression of mediators such as myokines, hepatokines or adipokines in each tissue to improve tissue-tissue interactions associated with obesity. A more comprehensive analysis may lead to the identification of such mediators that improve obesity pathophysiology by MC903.

There are several concerns about the clinical application of the present study. Previous clinical investigations reported that the serum concentration of VitD3 was negatively associated with body weight, fat volume, and insulin resistance [61, 62]. In addition, VitD3-deficient individuals have an increased risk of developing diabetes mellitus [22]. Moreover, VitD3 supplementation during resistance training has been reported to increase muscle quality in elder men, although cross sectional area of quadriceps did not change [63]. In contrast, the effects of VitD3 supplementation on glucose metabolism are inconsistent; some studies showed beneficial effects on glucose metabolism, but their cohort size was relatively small, whereas other studies only observed significant effects in a limited population, such as patients with pre-diabetes or low VitD3 levels, or no beneficial effects [21, 22]. Due to these inconsistencies, several large-scale clinical trials have been planned and are currently underway to elucidate the impact of VitD3 supplementation on obesity and glucose metabolism [22]. Unfortunately, an initial trial reported that VitD3 supplementation did not reduce the risk of diabetes among high-risk subjects examined without focusing VitD3 levels [64]. Further clinical trials are needed to clarify the clinical characteristics of subjects for whom VitD3 supplementation will be effective. In addition, there are several disorders for which VitD3 treatment is suggested to be effective, such as sarcopenia, non-alcoholic fatty liver disease, and Alzheimer’s disease [65–67]. We expect a transdermal treatment with a VitD3 analogue to become a new treatment option not only for diabetes, but also for many diseases for which the efficacy of VitD3-treatment has been established.

## Conclusions

A transdermal treatment with MC903 rapidly ameliorated obesity and associated metabolic dysregulations in diet-induced obese mice. Decreases in adipocyte sizes, increases in myofiber thickness and muscle *UCP*3 expression and improvements in energy metabolism were identified as the underlying mechanisms. Since the bioavailability of VitD3 by its oral administration is reduced in obese patients [40], the present study provides a new therapeutic approach involving the transdermal administration of MC903 for the treatment of obesity and diabetes.

## Supplementary Information

**Additional file 1: Figure S1**. Representative photomicrographs of H&E-stained sections of eWAT and an adipocyte size distribution analysis. **A** Representative photomicrograph of H&E-stained sections of eWAT. **B**, **C** Distribution histogram of adipocyte sizes in eWAT. Scale bar, 200 μm. **D** Weight of intrascapular brown adipose tissue (BAT). **E**, **F** Expression of Ucp1 in BAT or iWAT. **G** Representative photomicrographs of Oil Red O-stained of 3T3-L1 adipocytes differentiated with 300 nM VitD3 or MC903. Photomicrograph was taken at day 8 post-differentiation. Error bar, 200 μm. Data are presented as the mean ± S.E. N=6-8. *p<0.05, **p<0.01 significantly different from Chow mice

**Additional file 2: Figure S2.** Gene expression analysis of the gastrocnemius muscle in each mouse. Expression of myokine *Fndc5*, muscle differentiation-related genes, ubiquitin ligases, muscle fiber type-associated genes, and *vitamin D receptor* (*Vdr*) in the gastrocnemius muscle. Data are presented as the mean ± SEM. N=6-10

**Additional file 3: Figure S3.** Gene expression analysis of the soleus muscle in each mouse. The expression of myokine *Fndc5*, muscle differentiation-related genes, muscle degradation-related

## Data Availability

The datasets used during the present study are available from the corresponding author upon reasonable request.

## Abbreviations

AUC: Area under the curve
BAT: Brown adipose tissue
Emr1: EGF-like module-containing mucin-like hormone receptor-like 1
eWAT: Epidydimal white adipose tissue
Fbxo32: F-box protein 32
Fndc5: Fibronectin type III domain-containing protein 5
GTT: Glucose tolerance test
HFD: High-fat diet
Itgax: Integrin subunit alpha X
ITT: Insulin tolerance test
iWAT: Inguinal white adipose tissue
MRI: Magnetic resonance imaging
Mstn: Myostatin
Myf5: Myogenic factor 5
Myhc: Myosin heavy chain
Myod1: Myogenic differentiation 1
Myog: Myogenin
OCR: Oxygen consumption rate
Pgc1a: Peroxisome proliferator-activated receptor gamma coactivator 1-alpha
QR: Respiratory quotient
Tnfa: Tumor necrosis factor alpha
Trim63: Tripartite Motif Containing 63
UCP: Uncoupling protein
VDR: Vitamin D receptor
VitD3: Vitamin D3
VCO_2_: Carbon dioxide production
VO_2_: Oxygen consumption
