# Supplementary figures and images for: A transdermal treatment with MC903 ameliorates diet-induced obesity by reducing visceral fat and increasing myofiber thickness and energy consumption in mice
Source: Nutr Metab (Lond). 2023 Feb 11;20:10. doi: 10.1186/s12986-023-00732-5 (PMC9921322; doi:10.1186/s12986-023-00732-5)

# Supplemental Fig. 1

A

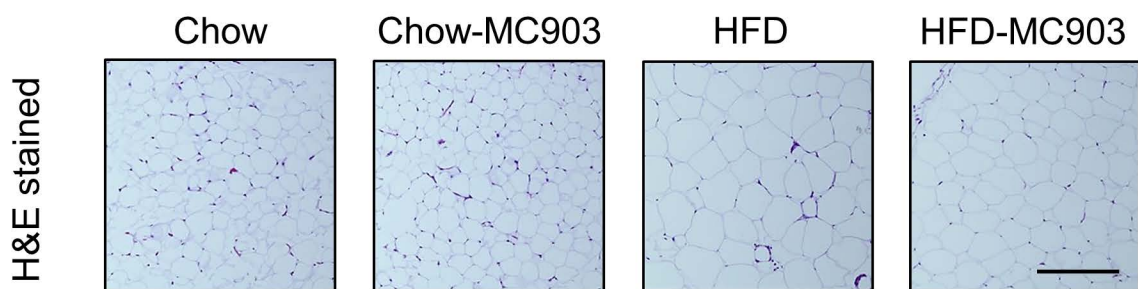

B

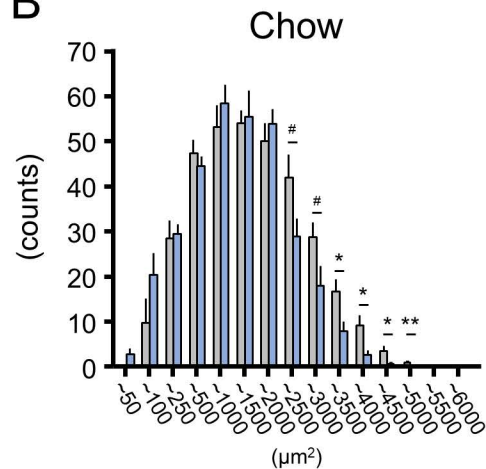

C

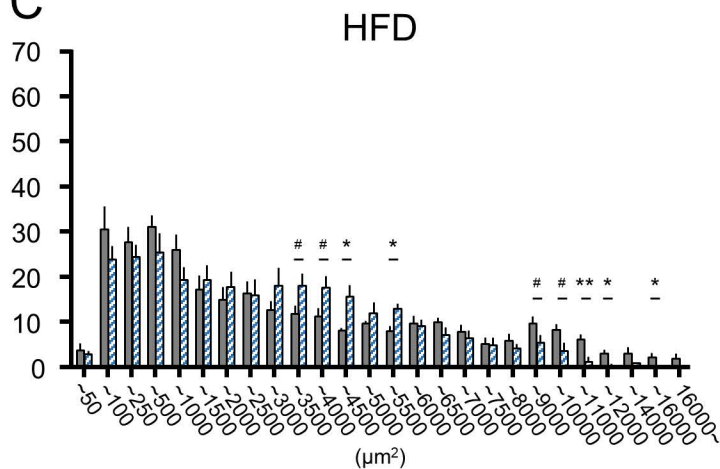

D

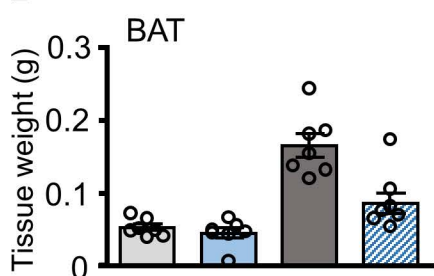

E

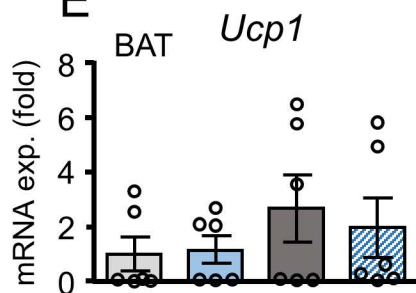

F

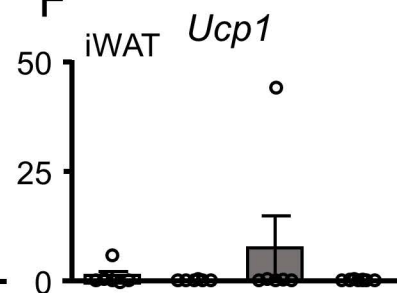

G

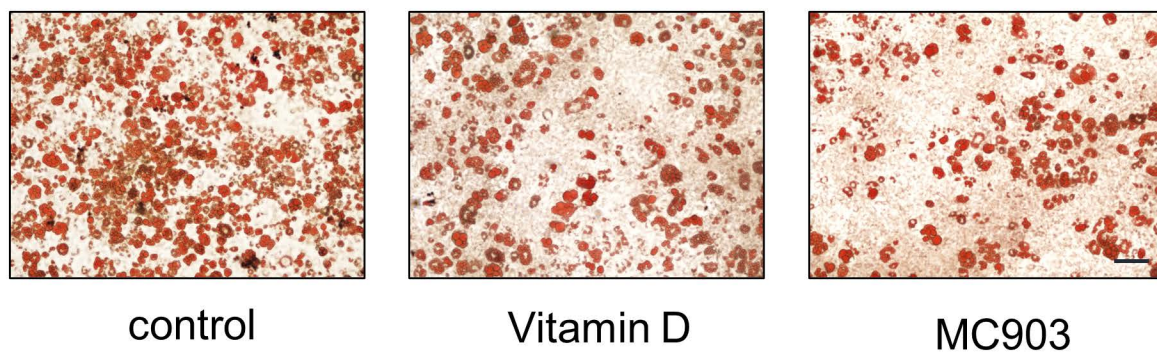

Chow Chow-MC903 HFD HFD-MC903

Supplement: Supplementary file 1 — Additional file 1: Figure S1. Representative photomicrographs of H&E-stained sections of eWAT and an adipocyte size distribution analysis. A Representative photomicrograph of H&E-stained sections of eWAT. B, C Distribution histogram of adipocyte sizes in eWAT. Scale bar, 200 μm. D Weight of intrascapular brown adipose tissue (BAT). E, F Expression of Ucp1 in BAT or iWAT. G Representative photomicrographs of Oil Red O-stained of 3T3-L1 adipocytes differentiated with 300 nM VitD3 or MC903. Photomicrograph was taken at day 8 post-differentiation. Error bar, 200 μm. Data are presented as the mean ± S.E. N=6-8. *p<0.05, **p<0.01 significantly different from Chow mice [file 12986_2023_732_MOESM1_ESM.pdf]

Supplemental Fig. 2

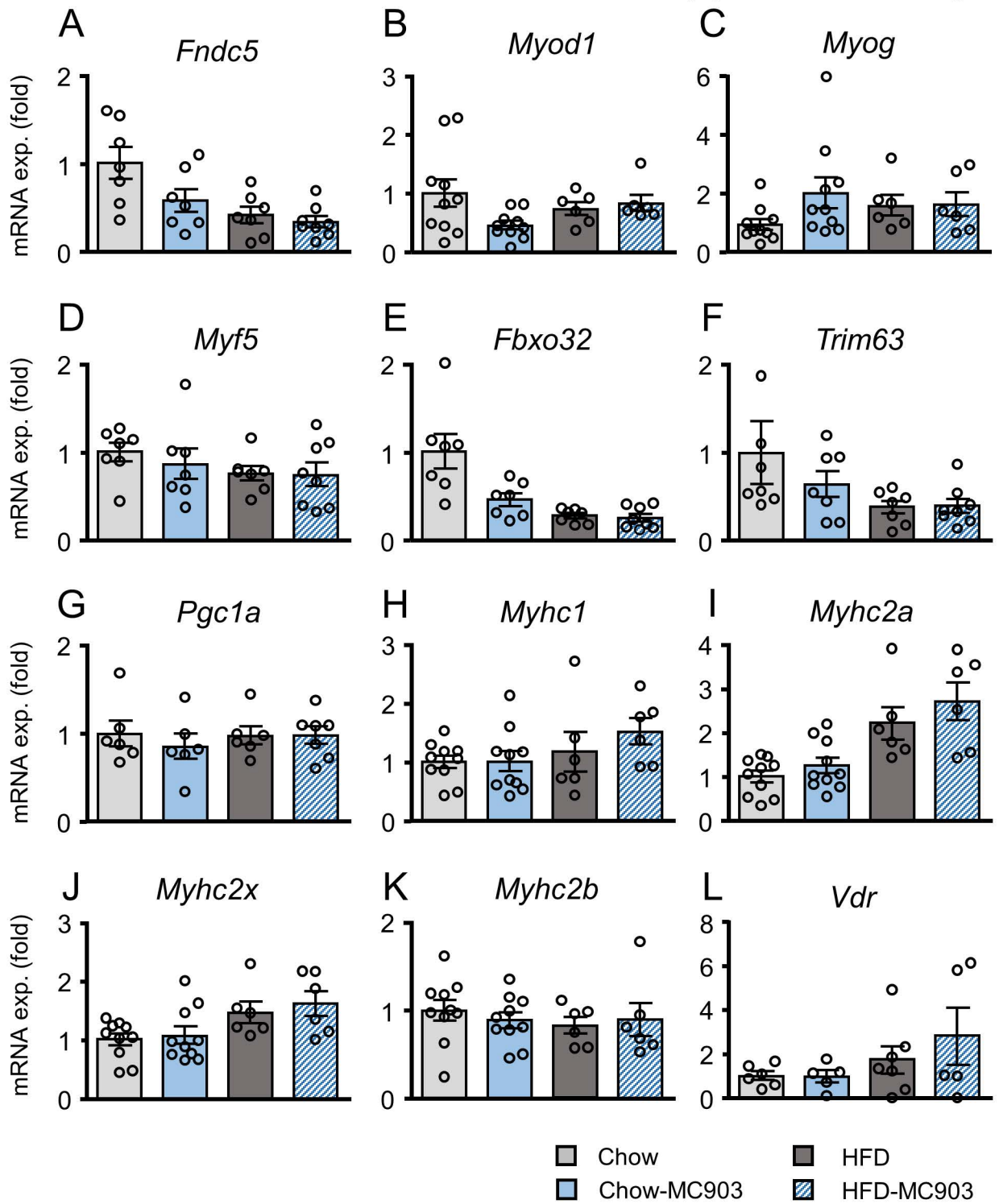

Supplement: Supplementary file 2 — Additional file 2: Figure S2. Gene expression analysis of the gastrocnemius muscle in each mouse. Expression of myokine Fndc5, muscle differentiation-related genes, ubiquitin ligases, muscle fiber type-associated genes, and vitamin D receptor (Vdr) in the gastrocnemius muscle. Data are presented as the mean ± SEM. N=6-10 [file 12986_2023_732_MOESM2_ESM.pdf]

Supplemental Fig. 3

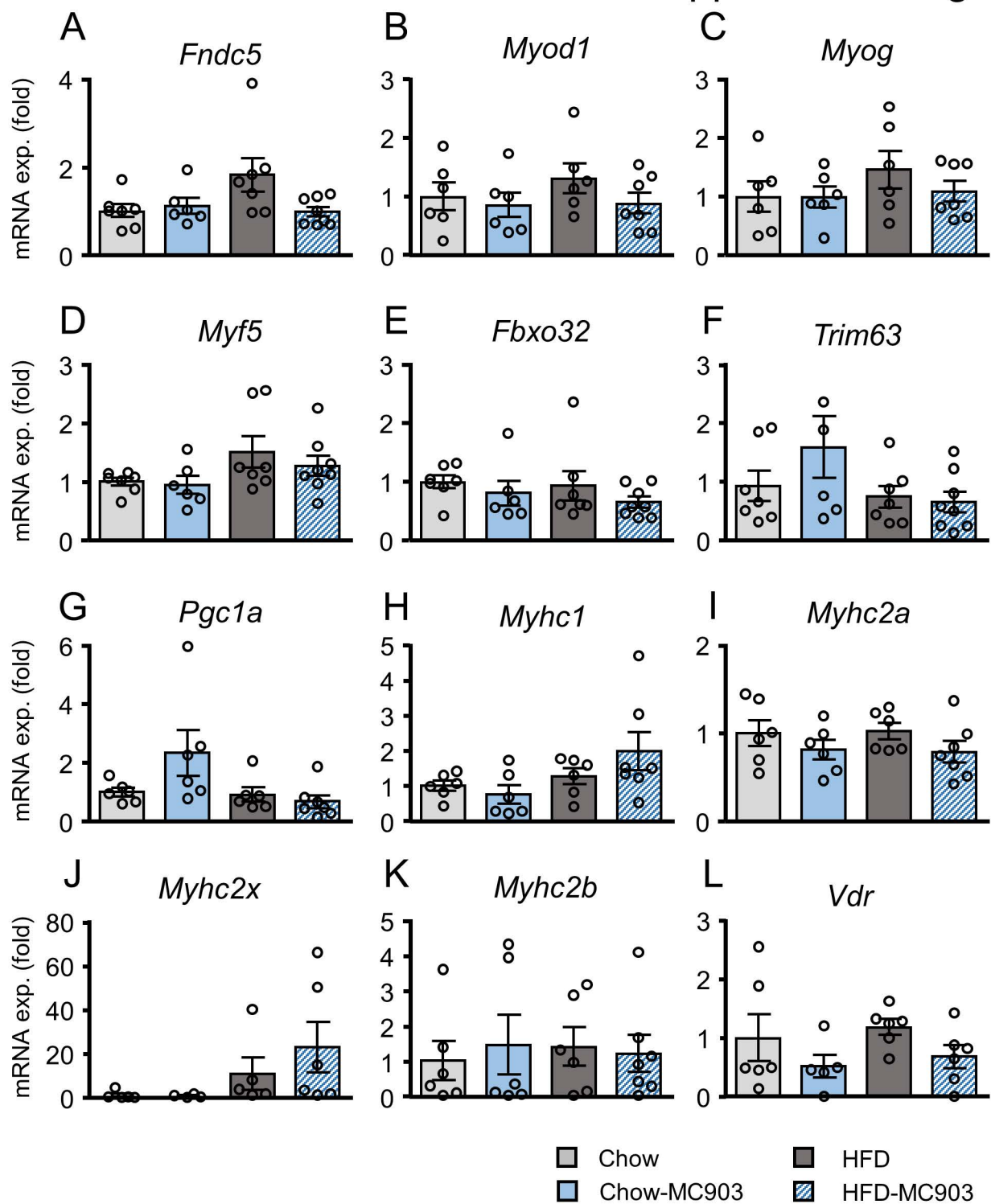

Supplement: Supplementary file 3 — Additional file 3: Figure S3. Gene expression analysis of the soleus muscle in each mouse. The expression of myokine Fndc5, muscle differentiation-related genes, muscle degradation-related [file 12986_2023_732_MOESM3_ESM.pdf]
